# Knowledge, practice and associated factors of infection prevention among healthcare workers in Debre Markos referral hospital, Northwest Ethiopia

**DOI:** 10.1186/s12913-018-3277-5

**Published:** 2018-06-18

**Authors:** Melaku Desta, Temesgen Ayenew, Nega Sitotaw, Nibretie Tegegne, Muluken Dires, Mulualem Getie

**Affiliations:** 1 Department of Midwifery, College of Health Science, Debre Markos University, PO. Box: 269, Debre Markos, Ethiopia; 2 Department of Nursing, College of Health Science, Debre Markos University, Debre Markos, Ethiopia; 3 Nursing health professional, Felegehiwot referral Hospital, Bahirdar, Amhara region Ethiopia; 4 Nursing health professional, Dejen Health center, Dejen, Amhara region Ethiopia; 5 Nursing health professional, Medawalebu university teaching hospital, Medawalebu, Oromia region Ethiopia

**Keywords:** Healthcare associated infection, Knowledge, Practice, Associated factors

## Abstract

**Background:**

Healthcare-associated infections are a major global public health agenda. Health care workers are front line of protecting themselves and clients from infection. This study examined the knowledge and practice of healthcare workers on infection prevention and its associated factors among health professionals working at Debre Markos Referral Hospital.

**Methods:**

A Hospital-based cross-sectional study was conducted with a structured pre-tested questionnaire among 150 participants. The healthcare workers were selected through systematic random sampling technique. Multivariate logistic regressions were computed to identify associated factors of knowledge and practice of infection prevention and variables with a *p*-value < 0.05 were considered statistically significant.

**Results:**

More than two thirds (84.7%) of healthcare workers were found to be knowledgeable but only 86 (57.3%) of respondents demonstrated a good practice on infection prevention. Older age, lengthy work experience and higher educational status were significantly associated with both knowledge and practice of infection prevention. In-service training, availability of infection prevention supplies and adherence to infection prevention guidelines was also associated with the practice of infection prevention.

**Conclusions:**

The finding of this study revealed a good knowledge of infection prevention on the majority of participants with relatively minimal practice rate. Sociodemographic factors and health facility factors were associated with knowledge and practice of infection prevention. Hospitals and other concerned stakeholders should ensure constant availability of guidelines and the provision of training to health providers. Moreover, developing professionals’ educational level, introducing infection prevention standard of practice and continuous mentorship was recommended.

**Electronic supplementary material:**

The online version of this article (10.1186/s12913-018-3277-5) contains supplementary material, which is available to authorized users.

## Background

Healthcare-acquired infections (HAIs) are a common global challenge mainly in low and middle- income countries [1]. An estimated 10% of hospitalized patients in developed countries and 25% in developing countries develop HAIs and subsequently results in adverse healthcare outcomes as increased hospital stay, economic burden, significant morbidity, and mortality. It is an unevenly distributed in developing countries, more than 90% of these infections occurred [2–4]. The high burden of HCAIs is due to lack of standardized infection prevention program, which was neglected due to limited resources, poor sanitary conditions and hygiene practices [5–8].

HCAIs are infections that were not present or incubating at the time of admission and are received by the patient during the process of care in a hospital or any other health care facility. Hepatitis B virus, Hepatitis C Virus, and HIV infection are commonest HAIs, mostly transmitted by healthcare workers who fail to practice infection prevention measures. Hence, Healthcare workers are front line of protecting themselves and clients from infection [9–13]. Infection prevention is a process of placing barrier between susceptible host and the microorganisms [14] and a major component of safe and high-quality service delivery at the facility level. Hence, HAIs assocaited morbidity and mortality are preventable through infection prevention strategy like, proper hand hygiene [15–19].

Implementing standard precautions like safety injection, isolation precautions (contact, droplet, and airborne precautions) [20], patient bathing [21], antibiotic stewardship, vaccinations, environmental cleaning, disinfection, and sterilization [22], comprehensive unit based safety program and surveillance were the major steps of infection prevention [23]. Surveillance data in real time allows infection control practitioners to identify and understand important nosocomial infections and to detect epidemics or outbreaks [24, 25].

There is an available low-cost intervention for infection prevention. Even though, the proportion of HCAIs are much higher in sub-Saharan Africa (18.9% in Mali, 14.8%, in Tanzania, 9.8% in Algeria [26–31] and 14.90%, in Ethiopia [6] and the majority of healthcare knowledge and adherence towards infection prevention strategies is still very low [32–37]. For this, improving the knowledge and practice of healthcare workers towards infection prevention is paramount to reduce the burden of HAIs.

In the resource constrained setting like many hospitals in Ethiopia, it’s difficult to control the infection rates of patients acquiring hospital infections and exposure of the HCWs to such infection. Some multi-targeted simple practical procedures that are part of the components of standard precautions against HCAIs and improving knowledge of infection prevention have been found to be effective in reducing the HCAIs. Despite, evidence regarding the level of knowledge and practice towards infection prevention and associated factors are not well explored in Ethiopia, including Amhara region [32–36].

In addition to this, there is no published data on the area of interest in the study area. Therefore, this study aimed to investigate the knowledge and practice of infection prevention and its associated factors of infection prevention among healthcare workers at Debre Markos hospital. The findings of the study will be used as an input for policy makers, programmers and health care workers to improve the clinical services and as well as a means of achieving Sustainable Development Goals.

## Methods

### Study design and setting

An institution based cross-sectional study was done from May 11–22,2015. The study was carried out at Debremarkos town, East Gojjam Zone, Amhara Regional state, Northwest of Ethiopia. Debremarkos referral hospital is located 295 km from Addis Ababa, the capital of Ethiopia and 265 km from Bahirdar, the capital city of Amhara regional state. Its astronomical location is 10^o^11’ North Latitude and 370 43′ East Latitude. The town has one government hospital and four health centers. Debre Markos hospital is one of the referral hospitals in Amhara Regional State and it potentially serves for more than five million people of the East Gojjam Zone and 4 districts of the West Gojjam Zone. The hospital has 286 clinical staffs, according to Debre Markos Referral Hospital human resource administration 3rd quarter report 2015.

### Study participants

All healthcare workers in Debre Markos Referal hospital were the source population. Selected healthcare workers who work at least 2 months in the direct care of patients in Debre Markos referral hospital in each ward of the hospital was the study population.

### Sample size determination and procedure

The sample size was calculated using single population proportion formula, n = (zα/2)^2^. p (1-p)/d2 by taking the proportion of good practice towards infection prevention activities 50%(since there was no previous study in the study areas). The following assumption was used; 95% confidence interval (CI) and 5% of marginal error. Considering 10% of contingency for non-responders, a total of 158 healthcare workers were included. Systematic random sampling was employed to identify the study population by using lists of health care workers posted in each ward of the hospital as a sampling frame. The first participant was selected randomly.

### Selection criteria

All health professionals who were working in selected health facility who have the qualification of doctors, health officers, midwives, nurses, x-ray technician, pharmacy and laboratory personnel who work at least 2 months in the direct care of patients in Debre Markos Referral Hospital in each ward of the hospital were included. Health workers who were seriously ill and on annual leave during data collection were excluded.

### Variables of the study and measurements

The dependent variables studied were knowledge and practice of healthcare workers towards infection prevention. Whereas, the independent variables include various sociodemographic characteristics (age, sex, marital status, religion, ethnicity, level of education, and work experience) and institutional factors (training about infection prevention, availability of infection prevention supplies). Knowledge about infection prevention was measured using the cumulative score of 10 questions each with two possible response [i.e. ‘’1 yes ‘’, 2 no].

Participants who have scored above the mean value for the cumulative score of knowledge questions were labeled as “Knowledgeable”. Likewise, fifteen questions were designed to assess participants practice regarding infection prevention.

Good practice: subjects answer above the mean score of practice assessment questions.

Adherence to infection prevention guideline: those healthcare workers who utilizes/used the available infection prevention guidelines/evidence/recommendations that reduce HAIs.

### Data collection and quality control

A self-administered questionnaire was used for data collection by distribution at the HCWs work unit and five diploma nurses were collect the data (Additional file 1). The tool was adapted from a modified CDC infection prevention and control assessment tool for acute care hospitals [38] and related kinds of literatures [32, 35, 36] and modified in our context. The questionnaire was prepared in English and translated into the local language (Amharic) and finally to English. Pre-tested was done in 5% of HCWs, in the study area, which was not included in the actual study to assess the content and approach of the questionnaire and necessary adjustments were made before actual data collection. The questionnaire was also tested for internal consistency (reliability) by Cronbach’s Alpha test and a score of 0.69 was obtained. The completeness, consistency, and accuracy of the collected data were examined by principal investigators on daily basis.

### Data processing and analysis

Data entry and statistical analysis was conducted by using SPSS versions (20.0). Summary statistics such as frequencies, proportions, the mean and standard deviation were computed. A bivariate and multivariate logistic regressions were employed between dependent and independent variables. The Knowledge score was dichotomized as 1 for knowledgeable, subjects answer above 50% mean score of knowledge assessment questions and 2 for not knowledgeable and practice score was also dichotomized as 1 for good practice and 2 for poor practice. Variables with a *p*-value of less than 0.2 in the bivariate analysis were then entered into a multivariable logistic regression to control effect of confounders. Model fitness (*p* = 0.25) and R squared of Cox & Snell and Nagelkerke (0.85) were determined. The statistical significance was declared at the *p*-value < 0.05 with 95% of Confidence interval (CI).

## Result

### Sociodemographic characteristics of the study participants

A total of 150 health professionals were interviewed yielding a response rate of 95% and majorities, 93(62%) were male. More than half of, 82(54.66%) were in the age group between 26 and 30 years old. The mean age of the respondents was 25.25 (SD ± 4.5) and majorities 92.66% of them were followers of Ethiopian Orthodox Christianity. A higher proportion (47%) of the respondents was diploma and 55.3% of healthcare worker were nurses (Table 1).

**Table 1:** Socio demographic characteristics of Debre Markos referral hospital health care workers in Debre Markos town, 2015

Variable		Frequency	Percentage%
Age	21–25	58	38.66%
	26–30	82	54.66%
	31 and above	10	6.66%
Sex	Male	93	62%
	Female	57	38%
Marital status	Single	85	56.66%
	Married	65	43.34%
Religion	Orthodox	139	92.66%
	Protestant	4	2.66%
	Muslim	7	4.66%
Educational status	Msc and above	20	13%
	BSc	60	40%
	Diploma	70	47%
Work experience	< 5 year	111	74%
	5–10 year	29	19.3%
	> 10 years	10	6.7%
Profession	Physician	21	14%
	Nurse	83	55.3%
	Midwifery	18	12%
	Health officer	3	2%
	Lab technician	13	8.7%
	Others^a^	12	8%
Had taken IP training	Yes	53	35.33%
	No	97	64.67%
IP guideline available	Yes	68	45.3%
	No	82	54.7%

^a^Emergency surgery, x-ray technicians, anesthetic provider, *IP* infection prevention

### Knowledge about infection prevention

The mean score of the knowledge questions was 5.29 (SD = 1.6). In this study, only 127(84.6%) [95% CI: 23.3, 30.5] of the respondents were found to be knowledgeable about infection prevention. Among the study respondents majority, 140 (93.33) and 141(94%) knew that disinfection and antiseptic prevent healthcare-acquired infection respectively. One hundred and thirty-two (88%) healthcare workers believed that every equipment needs decontamination before sterilization. More than half of the respondents (52%) haven’t known concerning the preparation formula for preparing 0.5% chlorine solution (Table 2).

**Table 2:** Knowledge of Debre Markos referral hospital health care workers in Debre Markos town, 2015

Variables	Level of knowledge	Frequency	%
Disinfection prevent health care acquired infections	Yes	140	93.33
	No	10	6.67
Antiseptic prevent health care acquired infection	Yes	141	94
	No	9	6
Chemical sterilization technique used for every equipment	Yes	58	37.31
	No	92	62.69
Physical sterilization (heat/radiation technique used for every equipment	Yes	50	37.31
	No	84	62.69
All microorganisms including spores are destructed by autoclaving	Yes	110	82.1
	No	24	17.9
Every equipment need decontamination before sterilization	Yes	132	88
	No	18	12
Protective device minimizes health care acquired infection	Yes	131	87.33
	No	19	12.67
Wearing gloves replace the need for hand washing	Yes	55	36.67
	No	95	63.33
preparing 0.5% chlorine solution	Yes	72	48
	No	78	52
There is PEP for HIV after exposure.	Yes	130	86.66
	No	20	13.34

*PEP* post exposure prophylaxi

### Practice of health care workers towards infection prevention

In this study, the proportion of healthcare workers who had good practice towards infection prevention activities was found to be 86(57.3%). Regarding of hand washing practice, 66 (44%) and 100(66.7%) of them were washing their hands with soap before patient care, after patient care or after contact with blood. Majority of the respondents hadn’t worn goggle 108 (72%) and 107(71.34) doesn’t vaccinate for the common pathogen. In regard to availability of Infection prevention supplies, 50(33.3%) of healthcare workers doesn’t use infection preventions supplies due to unable to get available supplies. Despite 38 (25%) of the healthcare provider who doesn’t use the available supplies due to being carelessness (70%) and 30% due to don’t perceiving exposure (Table 3).

**Table 3:** Practice of Debre Markos referral hospital health care workers on infection prevention Debre Markos town, 2015

Variables		Practice	
		Yes	No
Wash hands with soap before patient care		66 (44%)	84 (56%)
Wash hands with soap after patient care/contact with fluid		100(66.7%)	50(33.3%)
Wash hands without soap before and after patient care		70 (46.66)	80(53.34)
Used all type of personal protective equipment (PPE)		42(28%)	108(72%)
Type of PPE in patient care	gloves	128(85.33)	22(14.67%)
	goggles	140(93.3)	10(6.7%)
	mask	42(28%)	108 (72%)
	gown	62(41.33)	78(58.67%)
Changing time of chlorine solutions	Every 24 h	85(56.7%)	65(43.3%)
	After two days	45(30%)	105(70%)
	Don’t know	20(13%)	130(87%)
Used Infection prevention guideline/evidence		53(35.3%)	97(64.7%)
Recap needle before disposing		48 (32%))	102(68%)
History of contact for blood, fluid or stick injury		98(65.33)	52(34.67%)
Measures used after exposed for blood/stick injury (*n* = 98)	Taking PEP	59(60%)	39(40%)
	Clean by alcohol	70(71.5%)	28(28.5%)
	Washing with water	85(86.7%)	13(13.3%)
Give health education for patients about HCAI		97(64.66)	53(35.34%)
Cover wounds on the skin before you start your work		98(65.33)	52(34.67%)
Vaccinated against common pathogen		43(28.66)	107(71.34)
Used needles or sharps put on containers		97(64.66)	53(35.34%)
Containers disposed of when they are three quarters full		60(40%)	90(60%)

*HCAI* Health Care Acquired Infection, *IP* Infection prevention, *PEP* Post exposure prophylaxis

### Factors associated with knowledge of healthcare worker about infection prevention

In the bivariate analysis factors which were significantly associated with knowledge about infection prevention was: age, educational status, working experience, sex of the participants, profession and ever taking training in infection prevention methods. After controlling the confounding in multivariate logistic regression analysis, age, educational statuses, working experience, sex of the participants and ever taking training on infection prevention were found to be significantly associated with knowledge on infection prevention.

For thus, Healthcare workers whose age 31and above were about three times more Knowledgeable about infection prevention than when compared to those aged 21–25 (AOR = 3.15,95%, CI = [2.467–5.025]). Those male healthcare workers were two times more likely knowledgeable than those female healthcare workers (AOR = 2.05, 95%, CI = [2.139–5.816.

This study revealed that the working experience was found another strong predictor of knowledge towards infection prevention which shows that Healthcare workers who had work.

experience of above ten years was four times more likely knowledgeable on infection prevention than those had work experience of fewer than five years (AOR = 4.03, 95%, CI = [1.229–5.68]).]).

In regard to educational level, Healthcare workers with an educational level of Msc or above and were three times (AOR = 3.034, 95%, CI = [1.856–4.756]) and BSC were two times (AOR = 2.15, 95%, CI = [3.245–8.789]) more likely knowledgeable than Diplomas.

Furthermore, multiple regression showed, Healthcare professionals who haven’t taken Infection prevention training were75% less likely knowledgeable (AOR = 0.25, 95%, CI = [1.689–3.95) about infection prevention than those had taken training in infection prevention (Table 4).

**Table 4:** Bivariate and Multivariate analysis on assocaited factors towards knowledge of infection prevention among Debre Markos referral hospital health care workers, 2015

Variable		Knowledgeable		COR (95% CI)	AOR(95 CI)	*P*-value
		Yes	NO			
Age	21–25	48	10	1	1	
	26–30	73	19	3.218(1.787–5.793)*	0.144(0.037–3.555)	0.3
	31 and above	6	4	2.137(1.9–5.07)*	3.15(2.467–5.025)*	0.02*
Sex	Male	77	16	3.874(3.808,8.303)*	2.05(2.139–5.816)*	0.04*
	Female	50	7	1	1	
Marital status	Single	69	16	0.467(0.08–2.468)		
	Married	58	7	1		
Religion	Orthodox	118	21	2.57(0.073–3.345)		0.06
	Protestant	3	1	3.56(0.934–8.647)		0.08
	Muslim	6	1	1		
Educational status	Msc and above	20	0	4.24(1.39–6.89)*	3.034(1.856–4.756)*	0.01*
	Bsc	48	12	2.59(2.46–7.98)*	2.15(3.245–8.789)*	0.035*
	Diploma	59	11	1	1	
Work experience	< 5 year	94	17	1	1	
	5–10 year	23	6	5.467(0.134–6.567)	2.467(0.234–3.67)	0.05
	> 10 year	10	0	0.79(1.34–7.54)*	4.03(1.229–5.68)*	0.00*
Profession	Physician	20	0	1	1	
	Nurse	71	12	0.58(0.25–0.978)*	0.35(0.075–3.057)	0.3
	Midwifery	13	5	3.280(2.133–10.883)*	2.45(0.075–2.95)	0.2
	Health officier	2	1	2.874(0.808–5.303)		
	Lab technician	11	2	4.471(0.282–3.762)		
	Others*	10	2	6.45(0.758–2.895)		
Had taken IP training	Yes	52	1	1	1	
	No	75	22	2.56(3.68–6.98)*	0.25(1.689–3.95)*	0.045*
Available IP guideline	Yes	55	13	1		
	No	72	10	0.345(0.189–3.467)		

Others Emergency surgeon, x-ray technicians, anesthetic provider, *IP* Infection Prevention

* shows statistical significance at *p*-value < 0.05

### Factors associated with practice of healthcare worker on infection prevention

In the bivariate analysis, age, marital status, educational status, working experience, sex of the participants, availability of personal protective equipment and ever taking training on infection prevention methods were factors which were significantly associated with practice about infection prevention. However, age, educational statuses, working experience, ever taking training on infection prevention and availability of infection prevention supplies were found to be significantly associated in the multivariate analysis.

In respect to the age of healthcare workers, with the age range of 31and above were about two times more likely to practice infection prevention activities than those aged 21–25 (AOR = 2.04,95%, CI = [1.279–4.5793]). In regard to educational level, as the educational level increases the practice of infection prevention is increased based on this study. Multiple logistic regression of this study revealed that healthcare workers with an educational level of Msc or above were four times (AOR = 4.15, 95%, CI = [1.381–7.41]) more likely practice infection prevention activities than those healthcare works with diploma professionals and BSC holders were two times (AOR = 1.959, 95%, CI = [1.970–4.685]) more likely practiced infection prevention activities than those healthcare works with diploma professionals in respectively.

In addition, Healthcare workers who had work experience of above ten years had the highest odds of attaining infection prevention practice/activities than those who had work experience of fewer than five years (AOR = 3.17, 95%, CI = [1.98–5.674]). Healthcare workers who had taken infection prevention training were four times more likely to practice infection prevention than those haven’t taken training on infection prevention (AOR = 3.97, 95%, CI = [2.576–5.457]).

According to multiple regression analysis of this study, available supply of infection prevention increases the utilization of those supplies for the prevention of Hospital-acquired infections, Heath care works who get an available supply of infection prevention (as soap, mask, and infection prevention guideline) had higher odds of practiced infection prevention activities (AOR = 2.156, 95%. CI = [1.90–4.357]) than those healthcare works can’t get infection prevention supplies. Furthermore, adherence in IP guideline/evidence was another significant factor associated with the practice of infection prevention of HCAIs. Those healthcare workers who adhered to IP guidelines were four times more likely practiced infection prevention activities (AOR = 4.02, 95%, CI = [2.45–6.359] than those who doesn’t adhere to the guideline (Table 5).

**Table 5:** Bivariate and Multivariate analysis on assocaited factors of infection prevention practice among Debre Markos referral hospital health care workers, 2015

Variable		Practice		COR (95%CI)	AOR (95 CI)	*P*-value
		Good	Poor			
Age	21–25	33	25	1	1	
	26–30	47	35	2.643(0.961–3.947)	–	
	31 and above	6	4	3.53(2.67–5.89)*	2.04(1.279-4.579)*	0.02*
Sex	Male	52	41	2.450(0.183–6.722)	–	
	Female	34	23	1	–	
Marital status	Single	47	40	4.458(0.581–7.410)	–	
	Married	39	26	1	–	
Religion	Orthodox	77	62	3.573(0.371–8.347)	–	
	Protestant	3	1	2.750(0.183–16.722)	–	
	Muslim	6	1	1	–	
Educational status	Msc and above	14	14	3.346(2.567–5.872)*	4.15(1.381-7.41)*	0.001*
	Bsc	30	30	2.057(2.170–7.56)*	1.959(1.970-4.685)*	0.0038*
	Diploma	42	28	1	1	
Work experience	< 5 year	54	67	1	1	
	5–10 year	20	9	0.367(0.87–5.65)		
	> 10 year	9	1	2.47(2.98–7.256)	3.17(1.98–5.674) *	0.02*
Profession	Physician	11	10	1	1	
	Nurse	45	38	2.674(1.88–8.303)*	2.424(0.139-5.816)	0.85
	Midwifery	10	8	3.906(1.470–4.395)	1.25 (0.469–3.074)	0.32
	Health officers	2	1	1.571(0.282,4.762)	2.597(0.695–4.389)	0.09
	Lab technician	11	2	1.653(0.235–3.395)	0.786(0.967–5.28)	0.45
	Others*	7	2	2.425(1.075,5.8120)*	2.567(0.457-6.28)	
Had taken IP Training	Yes	42	11	2.63(1.26–5.95)*	3.97(2.576-5.457) *	0.008
	No	44	53	1	1	
Adequate supply of IP	Yes	62	38	1.79(1.358–6.53)*	2.158(1.90-4.357)*	0.01*
	No	24	26	1	1	
Adherence in IP guidelines	Yes	38	15	2.782(1.249–3.985)*	4.023(2.45–6.359)*	0.005*
	No	48	49	1	1	

*Statistical significance at *p* value < 0.05 and CI didn’t include 1with AOR

## Discussion

Infection prevention is one of the most important challenges in the health institutions. For this, the study assessed knowledge, practice and associated factors towards infection prevention among HCWs. In this study, the proportion of healthcare workers who were knowledgeable about infection prevention was found to be 84.7%.This finding indicated that majority of the healthcare workers in the hospitals had adequate knowledge on prevention of infections, a finding in line with many of similar and related studies in Zambia 74.4 [39] and Bahirdar city, 84.5% % [35]. This finding better than studies done in Nigerian, 65% [40], Nepal,22% [41], Palestine, 53.9% and Iran hospital, 57% (due to knowledge score difference) [42, 43] despite lower than a study done in Addis Abeba [32] and Dessie referral hospital, 95.7% [36]. This difference might be due to lack of in-service training, sample size, and sociodemographic difference.

The proportion of healthcare workers who were practicing proper infection prevention activities was 57.3% which in line with a study conducted in an Egyptian hospital [44] and in Bahirdar city [35]. However, this is much lower than studies [36, 41, 42]. This discrepancy might be due to a difference in knowledge of towards infection prevention, methodological, sample size, sociodemographic difference, lack of in-service training and infection prevention supply and professionals’ nonadherence to infection prevention.

This study revealed that healthcare workers with advanced age were significantly associated with knowledge (AOR = 3.15, 95% with CI of 2.467–5.025). This might be attributed to the fact that as the health care workers get older they are more likely advance their knowledge through experience and working with senior staffs. Male healthcare workers were found to be two times more likely to be knowledgeable about infection prevention when compared with females. The possible explanation of this finding might be linked with the educational status of participants as the majority of the BSc or Msc holders were males. This finding is in line with other studies [37, 42, 43].

Healthcare workers with higher educational level had more knowledge score than those who had a lower educational level. This might be so because healthcare workers with higher educational level might have acquired essential information, hence they might acquire infection prevention course [40, 42]. Lengthy of working experience was also another factor assocaited with knowledge score, which stated that health care workers who have served for more than 10 years were more likely knowledgeable on infection prevention. This is in line with findings from Ethiopia [35, 37]. This could be due to as the number of years of practice increases, health workers are exposed to repeatedly and became more experienced through working with senior staffs.

Furthermore, knowledge about infection prevention was significantly associated with ever taking training on infection prevention. Healthcare professionals who haven’t ever taken training less knowledgeable than counterparts. This is similar to studies on different countries [37, 40]. This might be the fact that those haven’t ever taken training would be less likely to get updated information, which hinders updating their knowledge on infection prevention.

Age is one of a significant factor of the practice of infection prevention, showed that healthcare workers who aged above 30 years or older were about two times more likely to practice infection prevention activities properly when compared with those who are less than 30 years old. This is comparable with other studies [37, 45]. This could be due to the fact as age advances, year of service increased which in turn improves their practice through time. In regard to educational level, healthcare workers with increased educational level were positively associated with a better practice of infection prevention activities than those healthcare works with lower educational level. This result is in conflict with a study done in Amhara region [37]. The difference might be due to sampling size, study participant difference and be misreporting or self-reporting.

In addition, this study revealed that working experience is another factor significantly associated with the practice of infection prevention activities. Health care workers who had work experience of above ten years were three times more likely practiced infection prevention activities which in line with a study in Bahirdar city [35]. Furthermore, in agreement with other studies [35, 46], this finding has shown that healthcare workers who had taken infection prevention training and get an available supply of infection prevention were more likely to have a good practice of infection prevention. The possible explanation for this finding could be the fact that training on current guidelines could upgrade the knowledge and skill of HCWs in that they would easily understand basic principles, standards of practice and implement them consistently. Besides this, up-to-date knowledge and skill regarding infection prevention could also increase the confidence of HCWs in complying with recommended guidelines and the available supply.

Moreover, this study showed that those healthcare workers who adhered the guideline were more likely practiced infection prevention activities than those who don’t adhere to the guideline. This is in line with other studies in Nigeria [40] and Australia [47]. This is due to the fact that those who adhered to the IP guidelines know the up-to-date information and perceive they are being exposed for HAIs, which improves their practice [46].

Despite extensive efforts have been made to minimize the possible shortcoming of this study, the finding could be interpreted in the presence of some inevitable limitations. The cross-sectional nature of this study will make it unable to form a temporal relationship between the outcome and predictor variables. The study is also prone to social desirability bias which could lead to over/underestimation of the study found.

## Conclusions

The study has demonstrated that majority of health care workers who had adequate knowledge about infection prevention and nearly above one-third of healthcare providers had poor practice towards infection prevention. Individual factors (advanced age, educational status, serving year, taking training and adherence on infection prevention and health facility factors were significantly associated with knowledge and practice of infection prevention.

In light of this finding, there is need to support existing and come up with new policies targeting these variables especially among the poor and vulnerable healthcare workers. Therefore, the Ministry of Health and the Hospital with the collaboration of other stake holders have to be made to update the knowledge and practice of health care workers regarding infection prevention activities with pre-service or in-service training, fulfilling necessary infection prevention supplies, developing of professionals educational level, introducing healthcare workers infection prevention standard of practice and continuous mentorship/supervision to improve HCWs adherence to infection prevention is recommended. Further Qualitative research on behavioral factors is also recommended.

## Additional file

Additional file 1: CDC Infection Prevention and Control Assessment Tool for Acute Care Hospitals, 2016. (DOCX 15 kb)

## Abbreviations

AOR: Adjusted Odds Ratio
BBP: Blood Borne Pathogen
COR: Crude Odds Ratio
HAIs: Healthcare associated infections
HCWs: HealthCare Workers
HIV: Human Immune Deficiency viruses
WHO: World Health Organization
