# Supplementary material for: Knowledge, practice and associated factors of infection prevention among healthcare workers in Debre Markos referral hospital, Northwest Ethiopia
Source: BMC Health Serv Res. 2018 Jun 18;18:465. doi: 10.1186/s12913-018-3277-5 (PMC6006704; doi:10.1186/s12913-018-3277-5)
Supplement: Supplementary file 1 — CDC Infection Prevention and Control Assessment Tool for Acute Care Hospitals, 2016. (DOCX 15 kb) [file 12913_2018_3277_MOESM1_ESM.docx]

**Supplementary file 1:** Assessment Practice of infection prevention in Ethiopia, using CDC Infection Prevention and Control Assessment Tool for Acute Care Hospitals, 2015

| **Assessment criteria** | **Response** | |
| --- | --- | --- |
|  | **Yes** | **No** |
| A.Hospital provides fiscal and human resource support for maintaining the infection prevention and control program. |  |  |
| B. Person(s) charged with directing the infection prevention and control program at the hospital is/are qualified and trained in infection control. |  |  |
| c. Written infection control policies and procedures are available, current, and based on evidence-based guidelines (e.g., CDC/HICPAC), regulations, or standards |  |  |
| D. Infection prevention and control program provides infection prevention education to patients, family members, and other caregivers. |  |  |
| E. Professional had taken a competency-based training program for hand hygiene or Personal protective equipment. |  |  |
| F. Supplies necessary for adherence to hand hygiene (e.g., soap, water, paper towels, alcohol-based hand rub) are readily accessible in patient care areas. |  |  |
| G. Hand hygiene policies promote preferential use of alcohol-based hand rub (ABHR) over soap and water in most clinical situations |  |  |
| H. Supplies necessary for adherence to personal protective equipment recommendations specified under Standard and Transmission-based Precautions (e.g., gloves, gowns, mouth,  eye, nose, and face protection) are available and located near point of use. |  |  |
| I. Adherence to preoperative surgical scrub and hand hygiene |  |  |
| J. Adherence to preoperative surgical scrub and hand hygiene |  |  |
| K. Appropriate use of surgical attire and drapes |  |  |
| L. Adherence to cleaning and disinfection of environmental surfaces |  |  |
| M. Adherence to hand hygiene |  |  |
| N. Adherence to infection prevention guideline |  |  |
| O. Scheduling of procedures allows sufficient time for all reprocessing steps |  |  |
| P. Injection safety ( recap of needle and presence of waste container ) |  |  |
| Q. prophylactic antibiotic selection based on procedure type. |  |  |

Available at [*https://www.cdc.gov/infectioncontrol/pdf/ICAR/Hospital.pdf*](https://www.cdc.gov/infectioncontrol/pdf/ICAR/Hospital.pdf)*.*
